# Knowledge-based DVH estimation and optimization for breast VMAT plans with and without avoidance sectors

**DOI:** 10.1186/s13014-022-02172-6

**Published:** 2022-12-06

**Authors:** Antonella Fogliata, Sara Parabicoli, Lucia Paganini, Giacomo Reggiori, Francesca Lobefalo, Luca Cozzi, Ciro Franzese, Davide Franceschini, Ruggero Spoto, Marta Scorsetti

**Affiliations:** 1 Radiotherapy and Radiosurgery Department, Humanitas Research Hospital IRCCS, Milan-Rozzano, Italy; 2 Department of Biomedical Sciences, Humanitas University, Milan-Pieve Emanuele, Italy

**Keywords:** RapidPlan, Knowledge-based planning, Avoidance sector, Breast, VMAT, DVH estimation model

## Abstract

**Background:**

To analyze RapidPlan knowledge-based models for DVH estimation of organs at risk from breast cancer VMAT plans presenting arc sectors en-face to the breast with zero dose rate, feature imposed during the optimization phase (avoidance sectors AS).

**Methods:**

CT datasets of twenty left breast patients in deep-inspiration breath-hold were selected. Two VMAT plans, PartArc and AvoidArc, were manually generated with double arcs from ~ 300 to ~ 160°, with the second having an AS en-face to the breast to avoid contralateral breast and lung direct irradiation. Two RapidPlan models were generated from the two plan sets. The two models were evaluated in a closed loop to assess the model performance on plans where the AS were selected or not in the optimization.

**Results:**

The PartArc plans model estimated DVHs comparable with the original plans. The AvoidArc plans model estimated a DVH pattern with two steps for the contralateral structures when the plan does not contain the AS selected in the optimization phase. This feature produced mean doses of the contralateral breast, averaged over all patients, of 0.4 ± 0.1 Gy, 0.6 ± 0.2 Gy, and 1.1 ± 0.2 Gy for the AvoidArc plan, AvoidArc model estimation, RapidPlan generated plan, respectively. The same figures for the contralateral lung were 0.3 ± 0.1 Gy, 1.6 ± 0.6 Gy, and 1.2 ± 0.5 Gy. The reason was found in the possible incorrect information extracted from the model training plans due to the lack of knowledge about the AS. Conversely, in the case of plans with AS set in the optimization generated with the same AvoidArc model, the estimated and resulting DVHs were comparable. Whenever the AvoidArc model was used to generate DVH estimation for a plan with AS, while the optimization was made on the plan without the AS, the optimizer evidentiated the limitation of a minimum dose rate of 0.2 MU/°, resulting in an increased dose to the contralateral structures respect to the estimation.

**Conclusions:**

The RapidPlan models for breast planning with VMAT can properly estimate organ at risk DVH. Attention has to be paid to the plan selection and usage for model training in the presence of avoidance sectors.

## Background

Breast cancer is one of the most commonly diagnosed tumours. It contributes conspicuously to the use of the resources of a radiotherapy department in all aspects, from diagnosis to treatment preparation, delivery and follow-up.

Over the last decade, many publications have proven the dosimetric advantages of using the volumetric modulated arc therapy (VMAT) technique for breast cancer treatment, particularly in terms of target dose homogeneity and conformity, and homolateral organs at risk (OAR) dose reduction [1–4]. However, its clinical application did not find the same diffusion as it did for most other anatomical sites. The main reason is related to the possible larger low-dose bath delivered with such a technique, with the associated increase of secondary cancer risk induction [5–7]. The conventional irradiation, with the classical two tangential fields, is the beam arrangement delivering the lowest dose to the contralateral structures (particularly breast and lung) since no direct beam crosses them. Such an arrangement allows these structures to receive only dose scattered from the primary treatment fields. Several studies approached different VMAT solutions, aiming to lower the dose to the contralateral structures. The classical VMAT arc geometry of about 240° long arc(s) has been modified over the years to solve the problem: a more tangential VMAT approach with one or two partial arcs covering all the directions from the classical medial entrance to an almost posterior position was implemented. The most explored technique reduced the breast en-face entrance, with the so-called “butterfly” or “bow-tie” technique, using short arcs mimicking a more tangential approach [8–19]. Other proposals have been hybrid techniques (VMAT and IMRT, or more common VMAT and 3DCRT) [20, 21] or non-coplanar settings [22–24].

The use of knowledge-based planning (KBP) allowed an improvement of the department's efficiency while maintaining (or leveraging) the quality of the treatment plans. RapidPlan (Varian Medical Systems, USA) is a KBP solution that allows implementing models based on departmental knowledge, bringing in the routine practice the plan quality according to the specific institutional goals. It allows developing DVH predictive models trained on high-quality historical plan data. These models can be used to prospectively estimate the DVHs of all the OAR included in the model training for any new patient with their anatomical characteristics. The RapidPlan estimations can then be translated into individualized optimization objectives based on the quality of the historical data used for the training [25–27].

The present study aims to analyze the combination of the RapidPlan models and the solutions from the VMAT optimizer implemented in the Eclipse planning system (Varian Medical Systems, Palo Alto, CA, USA) when the VMAT technique with an avoidance sector en-face to the treating breast is set. The work intends to understand possible weaknesses in the model configuration and its implementation in clinical practice.

## Methods

Twenty left breast patients were selected. Patients were simulated in the supine position with the arms above the head and in deep-inspiration breath-hold (DIBH) to better spare the heart.

The contoured structures included the lungs, the left Lung_Homolat and the right Lung_Contralat, the heart and the right breast (Breast_Contralat). The clinical target volume (CTV) was delineated to include the whole mammary gland. The PTV was defined as CTV plus an additional 5 mm margin, cropped inside the outer contour by 4 mm.

On each patient, two different plans were optimized for VMAT on a 6 MV (flattened) photon beam generated by a Varian TrueBeam linac equipped with Millennium MLC (5 mm leaf width at isocentre, in the central 20 cm of the field, 1 cm outside) to deliver 40.05 Gy in 15 fractions as mean dose to the PTV. The version 15.6.03 of the Eclipse treatment planning system was used. The Photon Optimizer (PO) engine and the Acuros algorithm were used for the plan optimization process and the final dose calculation, respectively.

Two sets of plans were generated for each patient:

- Plan *PartArc*: two or three partial arcs with the same start/end angles, starting from a classical medial entrance (around 300° gantry angle for the left-sided breast) and ending to a gantry value of approximately 160–170°. The collimator was set around ± 15° (complimentary), tuned on the target shape and patient anatomy. The X field size (direction of the MLC motion) was imposed to be less than 15 cm.
- Plan *AvoidArc*: the same as above, with an additional avoidance sector (arc sector with MU = 0 selectable inside the optimization window) defined in the optimization process for all the arcs. This tool forces the dose rate to drop to zero in the arc sector along which the contralateral structures (breast and lung) are visible, in the beam's-eye-view projection (the fields had the same size as the PartArc plan), beyond the target. The avoidance sectors thus prevent the primary beams’ irradiation of the contralateral structures.

The intention of AvoidArc plans thanks to the avoidance sector, is to maximally reduce the contralateral structures dose, going in that way to receive no dose from direct beams. The skin flash (procedure first proposed in [28]) was not adopted in this planning work to avoid confounding factors in the optimisation and in the calculation processes.

The generation of a RapidPlan DVH estimation model foresees an extraction and a training phase [25]. During the extraction phase, several anatomical and dosimetric features are obtained from the patient's anatomy and the plan. Each OAR is partitioned into sub-volumes according to its position in relation to the beam and the target. In the training phase, for the "in-field" OAR volume, a Principal Component Analysis (PCA) is conducted to find the geometric feature that best correlates with the dosimetric principal component score 1. With those two components, a regression model is applied to obtain the regression between the anatomical/geometrical and the dosimetric features. The OAR regions not belonging to the “in-field” category are modelled with simple models as means-and-std to estimate the dose. The final estimated DVH is a combination of the different parts according to the sub-volume partitioning.

To evaluate the models, the goodness-of-fit (the regression) is given as the coefficient of determination R^2^ and the average chi-square $\chi^{2}$. The first metric measures the proportion of the variance in the dependent variable (the dose, as the DVH principal component score) that is predictable from the independent variable (the geometrical feature). The second metric is related to Pearson's chi-squared test, which results from the residuals (the difference between the original and the estimated data). Also the potential outliers have to be evaluated. An outlier is an observation whose value is markedly different from the other values in the sample data. Among the metrics identifying a structure as a possible outlier, there is the modified Z-score (mZ), with a value greater than the threshold of 3.5. This metric measures the difference of an individual geometric parameter from the median value in the training set, normalized with the median absolute difference, and it is an indicator of potential geometric outliers.

The 20 PartArc plans were used to generate a RapidPlan model called *PartArcModel*, while the 20 AvoidArc plans were used to obtain a RapidPlan model called *AvoidArcModel*. The OARs considered in the models were the heart, lungs and contralateral breast.

The same optimization objectives were selected for both models, according to Table 1.

**Table 1:** Optimization objectives in the RapidPlan models

Structure	Parameter type	Objective	Priority
PTV	Upper objective	D_max,0%_ < 101% of prescription	95
	Lower objective	D_min,100%_ > 99% of prescription	95
Heart	Line objective	Generated	Generated
Lung_Homolat	Line objective	Generated	Generated
Lung_Contralat	Line objective	Generated	Generated
Breast_Contralat	Line objective	Generated	Generated

**Fig. 1:**
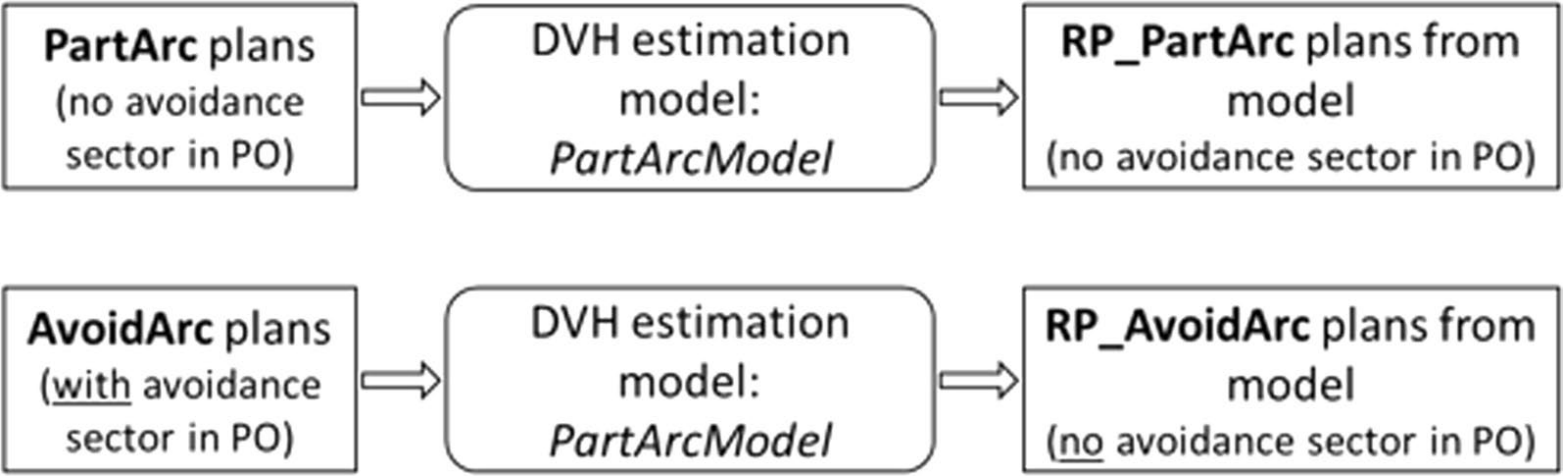
Diagram of the plans and models

The two models were then used to generate dose distributions on the same 20 patients executing the optimization process with the objectives determined by the model, without any human modification or interaction. Intermediate dose calculation was performed with Acuros during the optimization process to refine the dose accuracy. These test plans were optimized using the same plan geometry as the PartArc plans, i.e. without selecting the avoidance sectors inside the optimizer. The choice is supported by the fact that the arc geometry, as transferred to the model generation process, is technically the same in both PartArc and AvoidArc plans, as the beam setting does not include control point intensity information. This experiment was designed with two objectives. The first one was to verify if the optimization engine could autonomously reduce the dose rate in the sectors where the contralateral structures are seen in the beam's-eye-view beyond the target, using only the DVH estimation information translated into optimization objectives. The second objective was to analyze the ability of the model to generate the DVH estimations according to the AvoidArc dose distribution without the information on the avoidance sector's presence. For each patient, the two plans were named RP_PartArc and RP_AvoidArc, generated by PartArcModel and AvoidArcModel, respectively. The choice of the same set of patients in a closed-loop setting was intended to focus the attention on the model's capabilities by eliminating any possible variance induced by the use of different patients. This approach did not focus on the clinical application or the robustness of the models as done in an open-loop validation experiment. A schematic diagram of the plans and models is shown in Fig. 1.

The plans generated, as described above, were finally compared with the original plans used to train the two RapidPlan models. The comparison included a variety of dose-volume metrics. For the target V_95%_, V_105%_ and D_98%_ were scored. For the OARs, mean doses for the homolateral structures as well as V_20Gy_ for the left lung and V_18Gy_ for the heart, and the mean doses for the contralateral structures (right breast and lung) were used. A general focus of the planning efforts was to maximize the contralateral structures sparing.

Dosimetric data were obtained from the DVHs, while the statistical significance of the differences was tested with the Wilcoxon Signed Rank test, a paired non-parametric test; differences were considered significant with *p* < 0.05.

In the second part of the study, a RapidPlan model named *ButterflyModel* was generated from plans (called *Butterfly*) similar to the AvoidArc plans. These Butterfly plans were optimized according to a beam geometry obtained by splitting each arc into two short arcs instead of setting an avoidance sector in the optimization phase. No different collimator rotation was applied to mimic the AvoidArc plans fully. The motivation for this further step derives from the implementation of a similar VMAT technique for treating breast cancer in some radiotherapy centres (called "butterfly" or "bow-tie" techniques [8–19]). As expected, the differences between the AvoidArc and the Butterfly plans were minimal and insignificant.

The ButterflyModel was used to generate plans, named RP_Butterfly, having the same initial geometry as the Butterfly plans for subsequent comparisons.

## Results

### Plans PartArc and AvoidArc

Table 2 reports the dosimetric characteristics of the PartArc and AvoidArc plans used to generate the RapidPlan models. The different trade-offs are shown: for AvoidArc plans, a lower dose to the contralateral structures and the heart is achieved since no direct beam toward those structures contributes to their dose, while the contribution is only from the scattered radiation from the beams irradiating the target. The counter-balance is an increased dose to the homolateral lung and a slightly reduced, although significant, target dose homogeneity.

**Table 2:** Dose characteristics of the PartArc and AvoidArc plans selected for the models' input. Values are the average of the 20 patients cohort. Uncertainty is reported as one standard deviation

Structure	Dosimetric parameter and goal	PartArc plans	AvoidArc plans
PTV	V_95%_ > 95% [%]	97.0 ± 1.2	95.8 ± 0.6 ^+^
	V_105%_ < 3% [%]	0.7 ± 0.7	2.0 ± 0.8 ^+^
	D_98%_ > 90% [%]	94.1 ± 1.0	93.4 ± 0.6 ^+^
Heart	Mean < 4 Gy [Gy]	2.2 ± 0.4	1.9 ± 0.7 ^+^
	V_18Gy_ < 5% [%]	0.0 ± 0.0	0.1 ± 0.3
Lung_Homolat	Mean < 8 Gy [Gy]	5.4 ± 0.5	6.2 ± 0.8 ^+^
	V_20Gy_ < 10% [%]	6.9 ± 1.0	10.2 ± 2.0 ^+^
Lung_Contralat	Mean < 2 Gy (1 Gy)* [Gy]	1.4 ± 0.5	0.3 ± 0.1 ^+^
Breast_Contralat	Mean < 3 Gy (1 Gy)* [Gy]	1.7 ± 0.5	0.4 ± 0.1 ^+^

*the goal within brackets is in case of enhanced contralateral structures sparing

^+^*p* < *0.01 (Wilcoxon Signed Rank test)*

### The models

Table 3 summarises the model training results from the configuration information in terms of goodness-of-fit (coefficient of determination R^2^ and average chi-square $\chi^{2}$).

**Table 3:** Model training results

	Heart	Homolat lung	Contralat lung	Contral breast
*PartArcModel*				
Coeff. of determination R^2^	0.828	0.594	0.768	0.479
Average $\chi^{2}$	1.181	1.146	1.271	1.141
Number of Potential Outliers	1	5	10	1
*AvoidArcModel*				
Coeff. of determination R^2^	0.887	0.693	0.730	0.873
Average $\chi^{2}$	1.182	1.263	1.188	1.127
Number of Potential Outliers	2	1	5	6

In the table, the number of potential outliers is also reported. All the cases with mZ > 3.5 were evaluated in the single plans. None was judged to be a real outlier: the cohort of patients covered a range of possible anatomical differences.

**Fig. 2:**
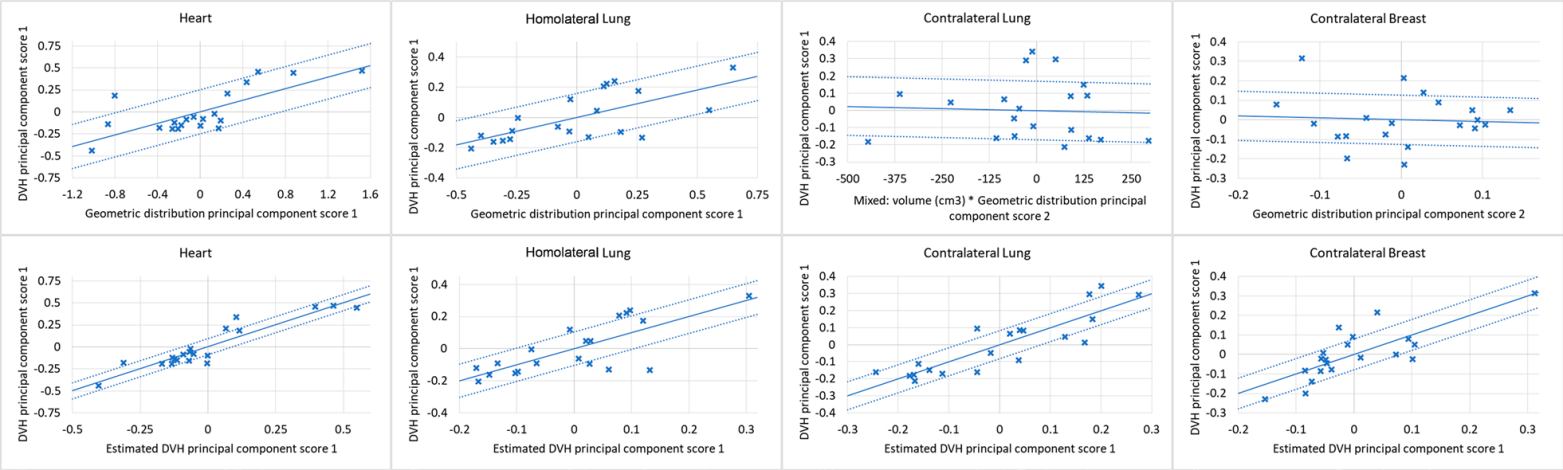
Regression plots (first row) and residual plots (second row) of the PartArcModel

**Fig. 3:**
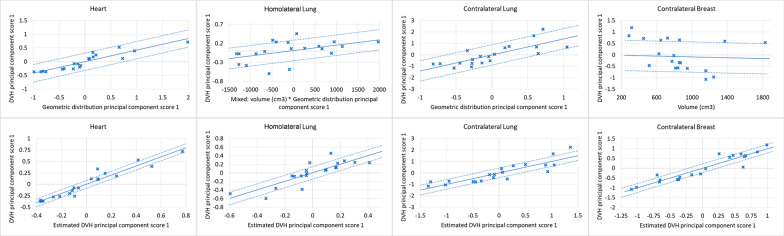
Regression plots (first row) and residual plots (second row) of the AvoidArcModel

The regression plots and the residual plots related to the four organs at risk are reported in Figs. 2 and 3 for the PartArcModel and AvoidArcModel, respectively.

Despite the contralateral breast for both models and the contralateral lung for the PartArcModel showed a regression plot without a clear slope and a large standard deviation (the dashed lines), the residual plots in the second row showed an accurate prediction with a small variance.

### Comparison

**Fig. 4:**
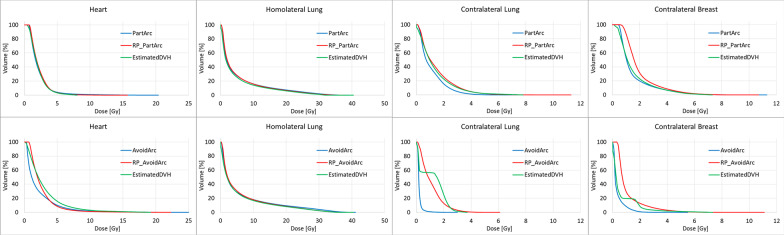
DVH of OARs comparison for Patient 8. First Row: PartArc (original plan), estimated DVH from PartArcModel, RP_PartArc (RapidPlan generated plan). Second Row: AvoidArc (original plan with avoidance sectors), estimated DVH from AvoidArcModel, RP_AvoidArc (RapidPlan generated plan, no avoidance sectors selected)

Figure 4 shows the DVHs of a single case (Patient8) for the four OARs of the original plans, the estimated DVHs from the RapidPlan models and the plans generated with the models: in the first row for the PartArc, in the second one for the AvoidArc. The other cases in the cohort present similar features to Patient8.

Table 4 reports the mean doses averaged over the patient cohort of the four OARs for the different plans and estimations.

**Table 4:** Mean doses (in Gy) for the organs at risk doses, uncertainty is reported as one standard deviation

	Heart	Homolat lung	Contralat breast	Contralat lung
PartArc	2.2 ± 0.4 Gy	5.4 ± 0.5 Gy	1.6 ± 0.5 Gy	1.4 ± 0.5 Gy
PartArcModel estimate	2.0 ± 0.4 Gy	4.8 ± 0.5 Gy	1.2 ± 0.4 Gy	1.3 ± 0.4 Gy
RP_PartArc	2.2 ± 0.5 Gy	5.5 ± 0.5 Gy	1.8 ± 0.4 Gy	1.4 ± 0.5 Gy
AvoidArc	1.9 ± 0.7 Gy	6.2 ± 0.8 Gy	0.4 ± 0.1 Gy	0.3 ± 0.1 Gy
AvoidArcModel estimate	2.7 ± 0.6 Gy	5.4 ± 0.6 Gy	0.6 ± 0.2 Gy	1.6 ± 0.6 Gy
RP_AvoidArc	2.6 ± 0.7 Gy	6.1 ± 0.6 Gy	1.1 ± 0.2 Gy	1.2 ± 0.5 Gy

In the first column, the plan generating the dose distribution

For the cases in which the avoidance sectors were not applied, the heart DVHs in the original plans, in the estimations and in the RP plans were similar, with an improvement of 3.6% (0.1 Gy) in the mean heart dose between the original PartArc and the RP_PartArc plans (*p* = 0.01). The model for the homolateral lung (low-density structure) resulted in similar DVHs, but the mean lung dose of RP_PartArc was higher by 2.8% (0.2 Gy) than PartArc (*p* = 0.003). However, the dose differences cannot be considered clinically significant. Mean doses to the contralateral structures were higher in RP_PartArc than PartArc (*p* = 0.01). Looking at Fig. 4, in RP_PartArc the optimizer seems unable to reduce the very low dose region of the contralateral breast, where the DVH estimation is consistent with the original PartArc plan. For the contralateral lung (low-density OAR), the DVH estimation is generally of lower quality, and the RP_PartArc is more consistent with the estimated objective.

In the cases with the avoidance sectors defined (AvoidArc), the homolateral lung model can well reproduce the original plan DVH (*p* = 0.28). At the same time, the mean heart dose is poorly estimated, and the resulting dose in the RP_AvoidArc plan increased by an average of 0.7 Gy (38%) with respect to AvoidArc. The situation of the contralateral structures is more complex. Table 4 reports the mean doses of RP_AvoidArc to the contralateral breast and lung, which is about 3 and 5 times higher than AvoidArc plans. In Fig. 4, the estimated DVHs present an unexpected shape (like a two-step DVH, where two parts of the structure volume receive different dose distributions), making the optimizer unable to spare those structures, as confirmed in Table 4. This unexpected shape shows that the training phase of the model configuration did not include information on the sectors with zero dose rate, leading to the inconsistency between the plans in the model (with the avoidance sectors) and the arc geometry (without the avoidance sectors).

**Fig. 5:**
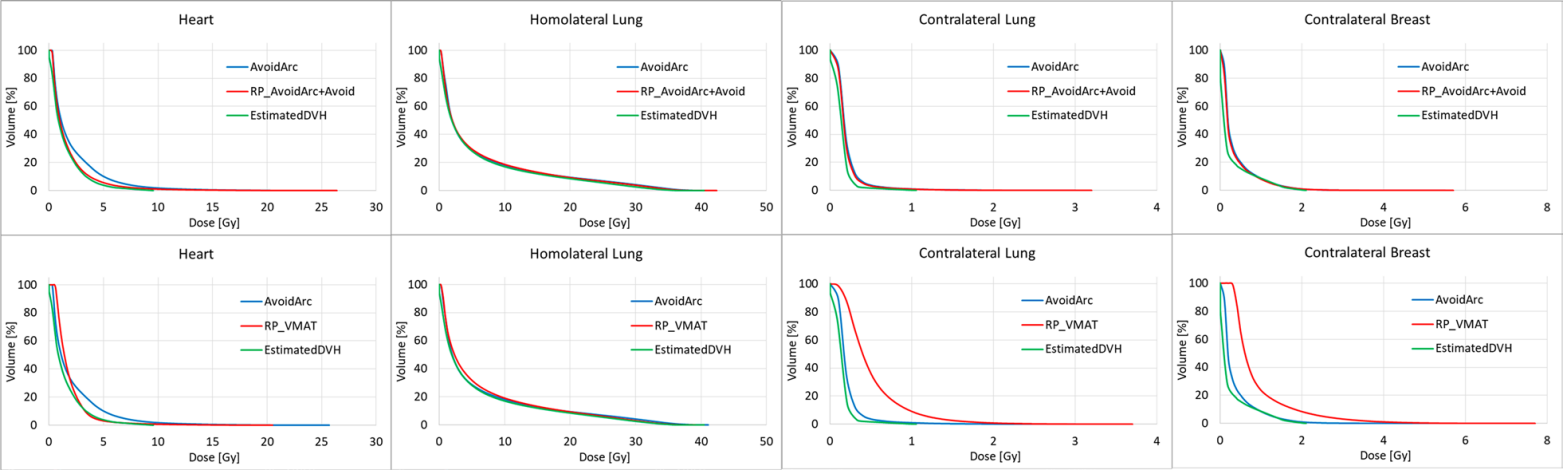
DVH of OARs comparison for Patient 8. First row: AvoidArc (original plan), estimated DVH from AvoidArcModel for a plan with avoidance sectors, RP_AvoidArc + Avoid (RapidPlan generated plan selecting an avoidance sector in the optimization phase). Second row: AvoidArc (original plan), estimated DVH from AvoidArcModel for a plan with avoidance sector, RP_VMAT (RapidPlan generated plan with no avoidance sector)

A set of new plans (RP_AvoidArc + Avoid) were optimized with the AvoidArcModel, setting the same avoidance sector in the optimization phase. The estimated DVHs in these cases reflect the presence of the avoidance sectors, leading to different estimates than those resulting from the RP_Avoid (where the avoidance sector was not defined). The first row of Fig. 5, likewise Fig. 3, reports the DVHs for the same Patient8 in this last condition. Here, the estimated DVHs for the contralateral structures presented the expected shape, consistent with the original AvoidArc plans, and the plans generated with the model reproduce the original ones. These results show the strong link between the model training and the beam geometry of the plans used in the model.

Finally, a set of plans, called RP_VMAT, with the PartArc geometry (no avoidance sectors defined) was optimized with the optimization objectives obtained for the RP_AvoidArc + Avoid plans. The results of this experiment are reported in the second row of Fig. 5 as DVHs for Patient 8 and in Table 5 as the mean dose to the OARs averaged over the whole patient cohort. The results show that the optimizer can lower the dose rate significantly in the direction toward the contralateral structures, although not down to zero, delivering some low doses to those structures that might be reduced by further decreasing the dose rate at some gantry directions.

**Table 5:** Mean dose (in Gy) to the organs at risk; uncertainty is expressed at one standard deviation

	Heart	Homolat lung	Contralat breast	Contralat lung
AvoidArc	1.9 ± 0.7 Gy	6.2 ± 0.8 Gy	0.4 ± 0.1 Gy	0.3 ± 0.1 Gy
AvoidArcModel estimate*	1.5 ± 0.7 Gy	5.5 ± 0.7 Gy	0.2 ± 0.1 Gy	0.2 ± 0.1 Gy
RP_AvoidArc + Avoid	1.8 ± 0.8 Gy	6.4 ± 0.9 Gy	0.4 ± 0.1 Gy	0.3 ± 0.1 Gy
RP_VMAT	2.0 ± 1.8 Gy	6.3 ± 0.8 Gy	0.9 ± 0.2 Gy	0.7 ± 0.2 Gy

In the first column, the plan generating the dose distribution

*with the avoidance sector defined in the optimization phase before the DVH estimation

### The ButterflyModel results

The results of the mean OARs dose of the Butterfly and RP_Butterfly plans are reported in Table 6. In this case, the avoidance sectors have been translated in the arc geometry, giving a consistency between the plans in the training and the model verification phase. RapidPlan gives here the expected results, outperforming, on average, the original plans (*p* < 0.01 for mean heart and contralateral breast doses, *p* = 0.014 for contralateral lung mean dose), except for the homolateral lung (not significant, *p* = 0.06).

**Table 6:** Mean dose (in Gy) to the organs at risk doses; uncertainty is expressed at one standard deviation

	Heart	Homolat lung	Contralat breast	Contralat lung
Butterfly	2.1 ± 0.8 Gy	6.2 ± 0.9 Gy	0.4 ± 0.1 Gy	0.3 ± 0.1 Gy
ButterflyModel estimate	1.7 ± 0.6 Gy	5.3 ± 0.9 Gy	0.2 ± 0.1 Gy	0.1 ± 0.1 Gy
RP_Butterfly	1.9 ± 0.8 Gy	6.3 ± 1.0 Gy	0.4 ± 0.1 Gy	0.3 ± 0.1 Gy

In the first column, the plan generating the dose distribution

## Discussion

The interest in the dose reduction to the contralateral structures has been widely explored in the last years, aiming to minimize the increased risk of secondary cancer induction due to the low dose bath. The classical VMAT (as PartArc) cannot reduce the low dose at the level of the tangential beam setting. Over the last decade, many authors described the VMAT solution with two short arcs mimicking the tangential treatment fields [8–12]. Fogliata et al. [10] and Rossi et al. [9] implemented this technique using avoidance sectors, as described in the present work. The VMAT approach of reducing the en-face entrance of the primary radiation has been proven to be the most beneficial solution, especially concerning the contralateral structure irradiation. Different authors compared this solution to the classical solution of the dual partial arc of about 200–250° each. The ratio of the mean doses to the contralateral breast and lung between the "butterfly" and the classical VMAT with continuous arc can be determined from different published works. From Pasler et al. [13], the ratios were 0.60 and 0.37 for the contralateral breast and lung, respectively.

Similarly, the results from Maier et al. [14] presented ratios of 0.72 and 0.59, 0.56 and 0.39 in the case of FFF beams; Xi et al. [15] reported data leading to ratios of 0.67 and 0.54; Viren et al. [16] of 0.46 and 0.56, Fogliata et al. [10] had ratios of 0.27 and 0.19, for breast and lung, respectively. This approach, reducing the mean dose to the contralateral structures, can also reduce the risk of secondary cancer induction, as proven by Fogliata et al. [7]. Other studies evaluating the same risk using VMAT for breast treatments showed an increased risk with the classical VMAT technique [5, 6], but in those studies, there was no specific attempt in the contralateral dose reduction, as in Paganetti et al. [6], where among the planning goal there was the mean dose to the contralateral breast to be less than 7 Gy.

Other solutions aiming to mimic the dose distribution of the tangential beam setting did focus on hybrid solutions with various combinations of tangential 3D conformal or IMRT beams with VMAT [20, 21]. These approaches outlined the difficulty of breast planning, which might be enhanced for particular anatomies, where the full VMAT solution improves the quality of the resulting doses [29].

The application of KBP planning with RapidPlan to breast treatments has been explored by different groups, particularly in the last few years [30–35], showing interest in such an approach.

It is clear that, since the best selection of the portion of the arc en-face the breast depends on the patient's anatomy, there is interest in benefitting from the predictive power of the knowledge-based planning models. The ideal scenario is to define a workflow where, starting from simple geometry (e.g. the classical continuous partial VMAT arcs), the model predicts DVH estimations, especially for the contralateral structures, and then can guide the optimization engine to find the best solution (e.g. zeroing the dose rate where needed) to achieve the clinical aims.

This study presented some criticalities related to this approach. On the one hand, we analyzed the ability of the RapidPlan models to estimate the DVH of structures receiving dose purely from scattered radiation with the avoidance sectors application. On the other hand, the study evaluated the ability of PO to optimize the plan toward the estimation in the case where the information of the zero dose rate sector is missing.

In the present study, as shown in the regression plots of the RapidPlan models, the contralateral structures did not present a clear correlation between the geometric and the dosimetric features, with an angular coefficient close to zero (Figs. 2 and 3). This might be related to the fact that the most significant component of the dose to those organs is the scattering. In such conditions, the dose estimation accuracy can be inferior in the structures far from the target. Spruijt et al. [36], discussing the out-of-field doses in breast irradiation with flattened and FFF beams, pointed out that there is an underestimation of the out-of-field dose calculated with the AAA algorithm (a type “b” algorithm). Considering the dose to the contralateral structures, the type of calculation algorithm should be taken into account because it could affect both the DVH estimation and the final dose calculation.

Moreover, for the DVH estimation, a non-negligible part of the contralateral organs is partitioned as an “out-of-field” region, where the means-and-std concept is used instead of the PCA-regression model, and the final DVH estimation is obtained as the relative sum of each region of the partition, weighted by the corresponding relative volume. In addition, the direct reconstruction from the principal component scores obtained from the regression model in these "extreme" conditions of the OAR far from the target could produce a curve that is not monotonically decreasing. This might also contribute to the unexpected estimated DVH shape of the contralateral structures, as shown in the second row of Fig. 4.

The mean doses of the contralateral structures (and in part of the heart), estimated by the AvoidArcModel, were higher than the corresponding mean doses of the input plans (AvoidArc). This is not the case for the homolateral lung and all the PartArc structures estimated by the PartArcModel, where the mean estimated dose is generally lower than the original plans. The inability of the AvoidArcModel to estimate mean doses to the contralateral OARs as low as those of the input plans derives from the missing information on the avoidance sector. The control points in that sector are used, during the model generation, to determine the OAR in-field region (where the PCA-regression model is used), while a large portion of the OAR volume should instead be managed as an out-of-field region, with the simplified mean-and-std model. The presence of the avoidance sectors in the original plans seems to determine an inconsistency in the data extraction phase. The RP_Butterfly cases instead show consistency between the geometric and the dosimetric information leading to a DVH estimation that is compatible with the actual beam arrangement (the sectors with zero dose rate in the AvoidArc plans are sectors with no beam in Butterfly and RP_Butterfly plans).

The case of RP_VMAT, where the avoidance sector was not included in the optimization while the optimization objectives did, is particularly interesting. Here the contralateral structures were better spared than the RP_AvoidArc plans, although not as much as in RP_AvoidArc + Avoid. The reasons for these differences could be a too low priority generated by the model, combined with the fact that PO does not reduce the dose rate below 0.2 MU/°, limiting the possibility of decreasing the contralateral doses.

The beam geometry influences the DVH estimation. A proof also comes from the mean doses estimated for the contralateral structures (where this fact is more pronounced) by the same AvoidArcModel in the two conditions of the avoidance sector defined or not in the optimization phase for the same initial beam geometry. In the first case, with the avoidance sector, the mean estimated doses to contralateral breast and lung were 0.4 and 0.3 Gy, respectively. Values to compare with 0.6 and 1.6 Gy, respectively, of the estimations in the case of no avoidance sector defined.

In summary, the model is able to produce good and consistent DVH estimations relative to the input data only in the case where the beam geometry information is correctly extracted and used in the model training or if the planner manually adjusts the beam geometry (avoidance sector), but this confirms the need of such information to the DVH estimator.

Regarding the homolateral structures, once the proper geometrical information is assigned, as in the PartArc or Butterfly cases, the plans generated with RapidPlan outperform the original plans only for the structure with soft tissue density (heart), while this is not the case for the low-density organ (lung), as shown for example in Table 4. This reduced performance in low-density structures is not generated by the RapidPlan process (the DVH estimation is, in fact, lower than the input plans), while it should be sought inside the optimization. A possible reason might be related to the fact that the irradiation of the lung presents an important scatter component. This is mainly in DIBH patients, as in this work, where the lung density is lower, as shown in [37], where the mean mass density is reported to reduce from 0.27 g/cm^3^ in free-breathing condition to 0.16 g/cm^3^ in DIBH. In such a case, the dose calculation algorithm used during the optimization iterations is not sufficiently accurate to account for scattering.

The Photon Optimizer in Eclipse has a tool, other than the avoidance sector, called *avoidance structure*. Those structures where the tool is activated will be shielded by closed MLC when the structure is before the target in the beam's-eye-view or whenever its projection is in the beam's-eye-view (as different options). However, due to MLC limitations, there could be some unexpected apertures or small beams irradiating the selected contralateral structures, and the transmitted dose increases the structure dose. For that reason, in the presented study, the used avoidance tool was in terms of the sector (with no radiation in a defined gantry rotation interval) instead of structure (with MLC closing toward the specified structures). However, it could also be of interest to evaluate a model generated by plans using that avoidance tool.

In this work, no skin flash was applied, although breast VMAT plans in the clinical practice have to include it by adding a virtual bolus (or body expansion) and optimizing on a target expanded inside the bolus to take into account the missing CTV to PTV margin in the skin direction. The rationale for not applying this important step was to evaluate the optimizer's ability to reduce the dose rate without confounding factors arising from the skin flash adoption. The clinical need for the skin flash in VMAT planning remains clear. The RapidPlan models used in clinical practice should be generated starting from plans with virtual bolus and target expansion.

The limited number of patients used in this study was sufficient for the scope of the work, which was not a clinical use of the presented models. It is important to mention that a model configured for clinical purposes should better include a larger number of patients adequately selected.

## Conclusion

In breast cancer treatments, the avoidance sectors can be used to limit the en-face beam entrance. In plans selected to train a RapidPlan model, this fact gives inconsistency between the dosimetric and the geometric data extracted for the model training process. The effect is a lower quality of the RapidPlan model.

The Photon Optimizer properly attempts to achieve the DVHs estimated by the RapidPlan models; however, it cannot reduce the dose rate below a threshold, thus limiting the possibility of reducing the dose to the contralateral structures as it would be done using the avoidance sectors.

## Data Availability

The information supporting this article's conclusions is included.

## Abbreviations

DVH: Dose-volume histogram
VMAT: Volumetric modulated arc therapy
CT: Computed tomography
AS: Avoidance sector
MU: Monitor unit
OAR: Organ at risk
DIBH: Deep-inspiration breath-hold
CTV: Clinical target volume
PTV: Planning target volume
MLC: Multi-leaf collimator
PCA: Principal component analysis
FFF: Flattening filter free
AAA: Anisotropic analytical algorithm
